# Leiomyoma of the Renal Vein: A Rare Tumor Presenting as a Renal Mass

**DOI:** 10.1155/2015/950584

**Published:** 2015-04-05

**Authors:** Cengiz Kocak, Sahin Kabay, Burak Isler

**Affiliations:** ^1^Department of Pathology, Faculty of Medicine, Dumlupinar University, Evliya Celebi Campus, 43000 Kutahya, Turkey; ^2^Department of Urology, Faculty of Medicine, Dumlupinar University, Kutahya, Turkey; ^3^Department of Urology, Evliya Celebi Education and Research Hospital, Dumlupinar University, Kutahya, Turkey

## Abstract

Leiomyomas are benign mesenchymal tumors that rarely occur in the kidney. Renal leiomyomas usually occur in the renal cortex or capsule. They are less commonly found in the muscularis propria of the renal pelvis and cortical vascular smooth muscle. In this case report, we present a 41-year-old woman who had right flank pain and detected a mass in the right kidney hilum.

## 1. Introduction

Renal leiomyomas are rare benign tumours of the kidney originating from smooth muscle cells [1]. Renal leiomyomas usually occur in the renal cortex or capsule [2]. They are less commonly found in the muscularis propria of the renal pelvis and cortical vascular smooth muscle [2]. Renal leiomyomas are classified into two major groups as small cortical and larger neoplasms. Small cortical or subcortical neoplasms are usually asymptomatic and are generally detected incidentally during autopsy or surgery. Larger neoplasms arise from the renal capsules or blood vessels [2, 3].

In this paper, we present a case of renal vein leiomyoma in a 41-year-old woman.

## 2. Case Presentation

**Figure 1:**
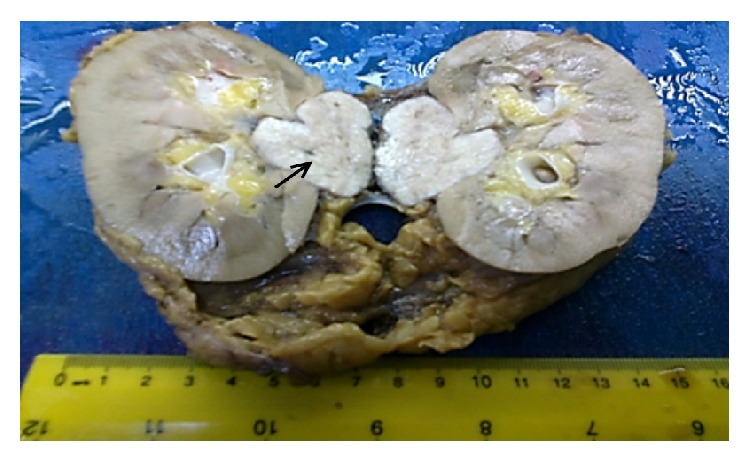
Gross specimen showing well-defined, lobulated tumor in relation to the right renal hilum.

**Figure 2:**
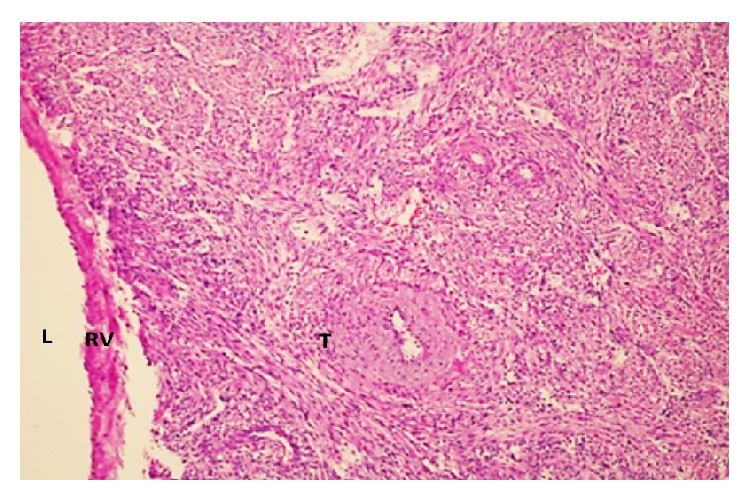
Histopathology slide shows cellular, nodular neoplasm consisting from benign smooth muscle cells with spindle shaped nuclei, eosinophilic cytoplasm (L: Lumen, RV: Renal vein, T: Tumour; H&E ×100).

**Figure 3:**
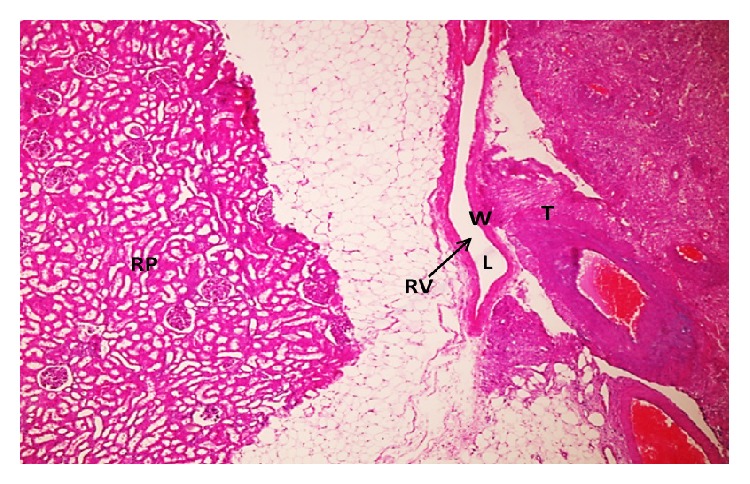
The relationship between tumoural tissue and renal vein (T: tumor, RV: renal vein, L: lumen, W: wall, and RP: renal parenchym; H&E ×40).

**Figure 4:**
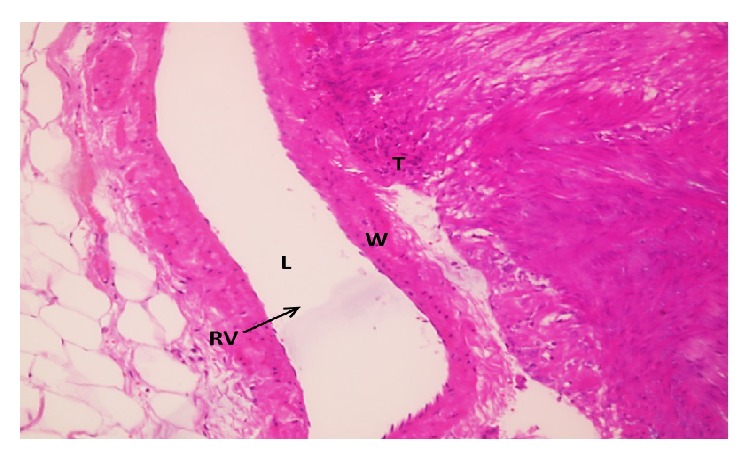
The relationship between tumoural tissue and renal vein (T: tumor, RV: renal vein, L: lumen, and W: wall; H&E ×40).

**Figure 5:**
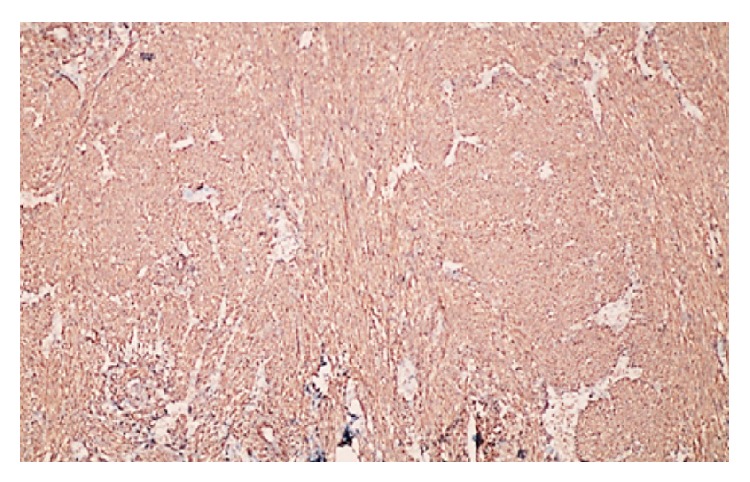
Diffuse SMA positivity in tumour cells (SMA ×40).

A 41-year-old woman was admitted to the department of urology with a complaint of right side pain; the history of the patient was unremarkable. Contrast-enhanced computed tomography (CECT) scan demonstrated a well-defined, lobulated mass lesion in the right kidney hilum, measuring 3.5 × 4.0 cm. Routine laboratory findings were within normal ranges. In view of the clinical and radiological features, prediagnosis of patient was considered as renal malign neoplasm. Laparotomy and right radical nephrectomy through left subcostal incision were made under general anesthesia. Macroscopic examination of the nephrectomy specimen revealed a tumour mass 4.0 × 3.5 × 3.0 cm in size attached to the right kidney at the hilum by a narrow isthmus of tumour (Figure 1). The tumour mass was well-defined, thinly encapsulated, and gray-white colored and demonstrated whorled pattern of smooth muscle bundles separated by connective tissue. Hemorrhage and necrotic foci and pericapsular or parenchymal spread were not seen. Biopsy samples were subjected to both pathology and immunohistochemical evaluation. Hematoxylin and eosin (H&E) staining revealed that the cellular, nodular neoplasm consisted of mesenchymal cells with spindle shaped nuclei, eosinophilic cytoplasm (Figure 2). Malignancy findings like cellular atypia, necrosis, and mitotic activity were not seen. The relationship between tumoural tissue and renal vein was observed in microscopic examination (Figures 3 and 4). Upon immunohistochemical examinations (Roche Ventana Benchmark-Bios, Arizona, USA, and Thermo Fisher Scientific Inc. MI, USA), while the neoplastic cells showed positive immunoreactivity for smooth muscle actin (SMA) (Figure 5), negative immunoreactivity was seen for S100, CD34, and human melanoma black 45 (HBM45). Considering all these features, the pathological diagnosis was leiomyoma originating from renal vein wall. 

## 3. Discussion

Leiomyoma was described by Virchow for the first time. Although leiomyoma can occur in every organ which contains smooth muscle tissue, it is frequently located in uterus. Genitourinary leiomyoma is most frequently located in the renal capsule [4]. On the other hand, primary vascular tumors are rare and usually present as smooth muscle tumors. They commonly arise from veins and vascular tumours are predominantly malignant. Benign vascular tumours as leiomyomas are very rare [5, 6]. Symptomatic renal leiomyoma is most frequently seen in 20–50-year-olds and females. The most common symptoms are palpable mass, pain, and hematuria [4, 7]. Diagnosis of renal leiomyoma is difficult with radiological examinations and diagnosis can be confused with renal cell carcinoma, oncocytoma, renal tubular adenoma, angiomyolipoma, and other malign tumours. Definitive diagnosis is established by histopathological examination after nephrectomy [3, 8]. Angiomyolipoma, leiomyosarcoma, schwannoma, and solitary fibrous tumour should be considered in histopathological differential diagnosis [8, 9]. In this case, we examined immunoreactivity of neoplastic cells for S100 to differentiate from schwannoma, CD34 to differentiate from solitary fibrous tumour, and HMB45 to differentiate angiomyolipoma. The neoplastic cells revealed negative immunoreactivity for S100, CD34, and HMB45. Angiomyolipoma includes adipose tissue and thick walled vessels as distinct from leiomyoma and demonstrates positive immunoreactivity for HMB45 [9, 10]. Schwannoma demonstrates positive immunoreactivity for S100. Solitary fibrous tumour demonstrates positive immunoreactivity for CD34. Marked cytologic atypia, mitosis, and necrosis are seen in leiomyosarcoma as distinct from leiomyoma [11].

After a thorough search of the literature, we could find only four references to leiomyoma arising in the wall of a renal vein [12–15]. Wells et al. reported a case of a leiomyoma arising from the wall of a right renal vein in a 75-year-old woman in 1981. The patient suffered from pain in the right side of abdomen. The intravenous pyelogram was normal. Laparotomy was performed and a lobulated mass was found anterior to the right renal pelvis surrounding the renal vein, running up into the renal hilum. Gross examination of the right nephrectomy specimen demonstrated a tumour mass measuring 9.0 by 4.5 by 4.5 cm attached to the right kidney at the hilum by a narrow isthmus of tumour [12]. Zelić et al. reported a patient with leiomyoma of the left renal vein in 2009. Tumour resection was performed by resecting a part of the vein along with the tumour and by ligation of the vein [13]. Kefeli et al. reported a 52-year-old man with leiomyoma of the left renal vein in 2009. The patient suffered from intermittent pain in the left side of abdomen during three years. CECT scan demonstrated a well-defined, homogenous solid mass lesion in the left renal hilar region, measuring 4 × 3 cm. Macroscopic examination of the left nephrectomy material revealed a tumour mass measuring 4 × 3 × 3 cm attached to the left kidney at the hilum [14]. Kumar et al. reported a case of leiomyoma of left renal vein in a postmenopausal woman that clinically resembled a retroperitoneal paraganglioma in 2014. The patient suffered from intermittent left hypochondrial pain. CECT scan showed a well-defined, lobulated, heterogeneously enhancing mass lesion in the left paraaortic region, measuring 3.6 × 3.5 cm. Left renal vein was invaded by the lesion with evidence of extension of tumor into left renal vein. Explorative laparotomy and excision of mass were made. A 3 × 4 cm soft tissue lobulated mass at the left renal hilum was identified. The mass was seen arising from the left renal vein and the renal artery was found to be adherent to the tumor. Left nephrectomy was done because of the complex location of the mass in the renal hilum. Histological features were consistent with renal vein leiomyoma [15]. This case is the fifth renal vein leiomyoma case in the literature according to our knowledge. Therefore, we thought that this case may be informative to the readers.

Consequently, in order to establish a definitive diagnosis of renal vein leiomyoma, careful histopathological examination of multiple sections has to be done.
